# Does timing of adjuvant chemotherapy influence the prognosis after early breast cancer?

**DOI:** 10.1038/sj.bjc.6603032

**Published:** 2006-03-07

**Authors:** C Alliot

**Affiliations:** 1 Hematology/Oncology Division, General Hospital of Annemasse, Annemasse, France. E-mail: alliotcfr@yahoo.fr

**Sir**,

I read with interest the article by Cold *et al* (2005) in the 19 September issue. The authors reviewed the database of the Danish Breast Cancer Cooperative Group (DBCG), including 7501 patients with early breast cancer who received adjuvant chemotherapy between 1977 and 1999. The patients have been divided into three groups according to the regimen, which were oral CMF (oral cyclophosphamide, methotrexate, 5-fluorouracil), intravenous CMF, and CEF (cyclophosphamide, epirubicin, 60 mg m^−2^, 5-fluorouracil) comprising 352, 6065, and 1084 patients, respectively. No significant benefit was observed in patients who had received the first cycle within 21 days after surgery comparatively to those who received it between 22–28 days, 29–35 days, or 36–89 days. Nevertheless, this study deserves some comments. Most of the patients have been treated before 1998 and historically, only patients with large tumours were eligible for chemotherapy. Given toxicity, most of the patients were young such as illustrated in the cohort treated with classical CMF. Thus, patients with a high risk of resistance to chemotherapy have been selected. In this line, we cannot agree with the argument that the NSABP-B28 trial has demonstrated no improvement in terms of disease-free survival since neoadjuvant chemotherapy has been initially developed to avoid mastectomy in patients with large tumours. Recently, the Aberdeen trial of sequential neoadjuvant chemotherapy with anthracyclins followed by docetaxel has clearly revealed a subpopulation resistant to both drugs (Smith *et al*, 2002). This phenomenon has been illustrated in the setting of preoperative chemotherapy of non-small-cell lung cancer in which the benefit is predominant in stages I and II (Depierre *et al*, 2002). Moreover, we must point to the fact that most of the Danish patients have received suboptimal regimen since it is widely admitted that CMF is inferior to three-drugs regimen including an anthracyclins (Early Breast Cancer Trialists Collaborative Group, 2005; Levine *et al*, 2005). The CEF60 regimen also is probably inferior to either CAF50 (cyclophosphamide, doxorubicin, 50 mg m^−2^, 5-fluorouracil) or CEF100 (Piccart *et al*, 2001; Bonneterre *et al*, 2005). Another point is the limit of the statistical analysis secondary to the segmentation of the database into three distinct studies according to the chemotherapy regimen. Thus, for example, no conclusion should be drawn from the small population of 352 patients treated with classical CMF. Moreover, the oestrogen-receptor status was unknown in a large fraction of this subpopulation. The subpopulation of patients treated with CEF also is relatively limited. The comparison between perioperative chemotherapy and adjuvant chemotherapy might be discussed since the tumour cell populations probably are different in the two situations. The authors point that the benefit obtained by perioperative chemotherapy seems to disappear when patients receive classical adjuvant chemotherapy (van der Hage *et al*, 2001). This opinion might result from a misinterpretation of the experimental studies conducted during the 80s. In murine models, removal of a primary tumour increases the labelling index and angiogenesis of the distant micrometastases (Fisher *et al*, 1983; Folkman, 1990). The crucial data revealed by these studies are the timing of these mechanisms, which principally occur from D1 to D3 after surgery, then decrease rapidly from D4 to D7. Thus, the delay of 21 days is purely arbitrary and is supported by no biological argument. A meta-analysis of five trials using the suboptimal CMF regimen administered within 72 h after surgery has demonstrated an absolute benefit of 3.4% in terms of disease-free survival for patients treated within this delay (Clahsen *et al*, 1997). Moreover, another fundamental data that has been omitted by the authors are the release of circulating cells by tumour removal (Brown *et al*, 1995). In this line, we also might address the question of the possible release of circulating cells during biopsy, which could lead to a considerable increase in the delay to systemic therapy. After dissemination, the prognosis might be compromised since Braun *et al* (2000) have demonstrated that adjuvant administration of neither anthracyclins nor taxanes can eradicate micrometastases in the bone marrow. Resistance can be due to molecular mechanisms such as multidrug resistance but also by cell dormancy. Moreover, the stromal environment might promote tumour growth and resistance to chemotherapy as demonstrated in multiple myeloma (Nefedova *et al*, 2003). Thus, comparison between perioperative and adjuvant probably is not pertinent. Finally, perioperative chemotherapy should not be dissociated from neoadjuvant chemotherapy, which can be a predictive factor of response to our increasing number of active drugs. In conclusion, this study might support a confusion by comparing the optimal delay of perioperative chemotherapy, which tends to optimise systemic therapy, and the tolerable delay of classical adjuvant therapy, which evaluates the impact of limitations in medical resources (Altundağ *et al*, 2000).
